# Perforated Marginal Ulcer

**DOI:** 10.7759/cureus.38127

**Published:** 2023-04-25

**Authors:** Frederick Tiesenga, Luis F Adorno, Datiobong Udoeyop, Victor Dinh, Sarosh Ahmed, Akash Sharma, Karan Sharma

**Affiliations:** 1 General Surgery, West Suburban Medical Center, Chicago, USA; 2 Surgery, Windsor University School of Medicine, Community First Medical Center, Chicago, USA; 3 Surgery, Community First Medical Center, Chicago, USA; 4 Surgery, Windsor University School of Medicine, Chicago, USA

**Keywords:** roux-en-y gastric bypass (rygb), exploratory laparotomy, perforated viscous, gastric ulceration, duodenal ulceration, duodenal perforation, marginal ulcer perforation, marginal ulcer, duodenal ulcers, perforated duodenal ulcer

## Abstract

Marginal ulcers are a late complication of gastric bypass surgery. A marginal ulcer is a term for ulcers that develop at the margins of a gastrojejunostomy, primarily on the jejunal side. A perforated ulcer involves the entire thickness of an organ, creating an opening on both surfaces. We will present an intriguing case of a 59-year-old Caucasian female who arrived at the emergency department with diffused chest and abdominal pain that began in her left shoulder and went down to the right lower quadrant area. The patient was in visible pain with restlessness, and her abdomen was moderately distended. The computed tomography (CT) showed possible perforation in the gastric bypass surgery area, but the results were inconclusive. The patient had laparoscopic cholecystectomy ten days prior, and the pain began right after surgery. The patient underwent an open abdominal exploratory surgery, with the closure of the perforated marginal ulcer. The fact that the patient had undergone another surgery and had pain immediately afterward also obscured the diagnosis. This case shows the rare presentation of the patientäs diverse signs and symptoms and inconclusive reports that led to the open abdominal exploratory surgery that finally confirmed the diagnosis. This case highlights the importance of a thorough past medical history, including surgical history. The past surgical history led the team to zone in on the gastric bypass area, leading to an accurate differential diagnosis.

## Introduction

Marginal ulcers (MU) are a common complication after gastric bypass surgery, with an incidence between one and sixteen percent [1]. Smoking and non-steroidal anti-inflammatory drug use are the most common causes of ulceration, and late anastomotic ulcers can result in acute perforation and severe bleeding. In most cases, the ulcers respond to pharmacologic therapy and require no further intervention; however, in cases of perforation, surgical intervention is required using one of several repair methods, including omental patch repair [2]. Anastomotic ulcers typically present a year after the gastric bypass procedure [3]. We present a case of pneumoperitonitis due to a perforated marginal ulcer and the surgical intervention required to treat it.

## Case presentation

**Figure 1:**
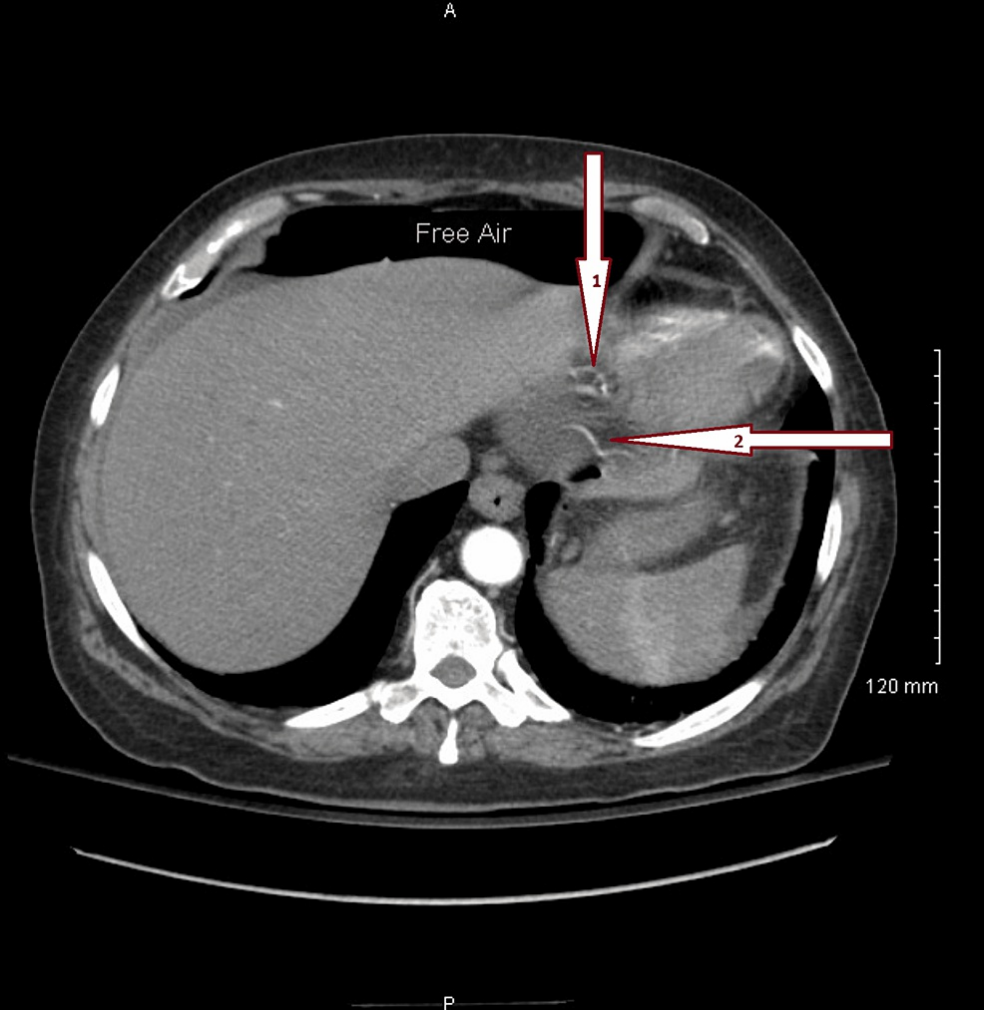
Abdominal CT where possible ulceration occurred (pylorus) Important to note that the black shade above the liver is free air. The free air was an indication that there was a perforation. Arrow 1 from the top is the area where said perforation occurred. Arrow 2 points to where possible perforation might have occurred.

A 59-year-old female presented to the emergency department with complaints of left shoulder pain that radiated down to her abdomen. The patient was anxious and visibly in pain, and her abdomen was mildly distended. Abdominal guarding was present and showed signs of severe pain to light palpation. The patient described the pain as "something exploded inside her". The patient smoked and drank alcohol socially for the past 21 years, with a medical history of atrial fibrillation since 2019 and hypertrophic cardiomyopathy. She had no known drug or medical allergies. The patient takes metoprolol (5mg) and hydrocodone. Her surgical history included a cesarean delivery, a gastric bypass performed in October 2021, and a cholecystectomy. Specifically, her cholecystectomy was performed on August 2022, ten days before arriving in the emergency department. She was stable in postop laparoscopic cholecystectomy; however, the pain worsened the next day. The computed tomography (CT) scan showed possible pathological free air and the presence of fluid in the abdomen (Figure 1). Due to the worsening pain and the CT results, she was scheduled for an exploratory laparotomy with possible intervention and repair.

Treatment 

The patient was placed under nothing by mouth (NPO) 12 hours before surgery, taken to the operative suite, and placed on general anesthesia for an exploratory laparotomy. A broad upper midline incision was used to open the abdomen during the procedure. Upon accessing the peritoneal cavity, there was a murky fluid in the upper abdomen, and cultures of the fluid were taken. Instantly, a perforated anterior marginal ulcer was recognized at the gastrojejunostomy. The ulcer was repaired with a Graham patch, and two Jackson-Pratt (JP) surgical drains were placed in the abdomen.

The patient complained of right lower quadrant and back pain post-surgery and was given fentanyl. The patient stated that fentanyl was not working, and the pain medication was changed to Dilaudid every three hours. The patient was placed on NPO for five days following the procedure. JP drain was removed on day five, and the patient was discharged with instructions to follow up in the clinic and to make a follow-up appointment with a gastrointestinal (GI) doctor. The patient was discharged with a clear liquid diet for two weeks, following another two weeks of a mashed diet.

## Discussion

Roux-en-y gastric bypass has widely been accepted as the gold-standard procedure for weight loss for patients with a BMI of 40 or greater or 35 in the presence of obesity-related comorbidities. The procedure provides 65% average weight loss for most patients, and about 50% of the primary excess weight loss is maintained in 85% of patients [4]. The documented mortality rate from the procedure is significantly low - about 0.1 to 0.3% - across various studies, but incidents of long-term complications, including marginal ulceration, at an average of 0.6% to 16% [5]. Subsequent perforation of said marginal ulcer, as discussed in this case review, is even rarer at an average rate of 0.7%. Documented risk factors, which we will discuss here, significantly increase the risk of developing perforated marginal ulceration.

Marginal ulcers have been recognized as a known post-operative complication following gastric bypass surgery. Several risk factors have been identified to be associated with the pathogenesis of MU in these patients. In an international survey by Steinemann et al. [6], they subdivided these risk factors into surgical and non-surgical. The survey identified the most influential surgical risk factors as ischemia of the small vessels and anastomotic tension. Less predominant, but still noteworthy, was the use of nonabsorbable sutures in reinforcing stapled gastrojejunostomy (GJ) and increased acid content in the gastric pouch. Smoking, *Helicobacter pylori (H. pylori​​​​​​​) *infection, corticosteroid use, and non-steroidal anti-inflammatory drugs (NSAIDs) were among the top non-surgical risk factors identified for the subsequent development of marginal ulcers. In the social history collection for the patient reviewed in this case study, she identified as a social smoker for the last 21 years. This is consistent with the increased risk of developing a perforated marginal ulcer postop.

*H. pylori *as a risk factor for the development of marginal ulcers postop gastric bypass is relatively controllable. The disease can be screened for and treated preop, and eradicating *H. pylori* is somewhat effective and cost-efficient. Although the incidence of *H. pylori *is directly increased in a patient who ends up developing MU postop, it has been questioned if eradication of it can indeed reduce the risk of developing MU postop since the increased risk remained even after eradication. The multifactorial etiology of MU still bears more significance than the isolation of *H. pylori*. In Steinemann et al.'s international survey [6], most surgeons admitted that they usually screen for *H. pylori* as part of the preop process and administer treatment to eliminate the infection when the results are positive. The patient in this case study presented with left shoulder pain radiating to the abdomen, which is not definitive for the diagnosis of MU. But, combined with the computed tomography (CT), which showed the presence of fluid in the abdomen and pelvis, possibly pathologic free air, it was highly suspected (Figure 1).

The diagnosis was confirmed after exploratory surgery, and the perforated anterior marginal ulcer was identified at the gastrojejunostomy. Felix et al., in their analysis, concluded that the average time when MU perforation was seen was at 18 months, with a range of three to 70 months [7]. The time range is consistent with our case study since the patient's symptoms occurred almost ten months postop. Felix et al. also agreed that smoking significantly increases the risk of marginal ulcer perforation in these patients [7]. Regardless of the identifiable risk factors associated with the development of MU, surgeons must quickly identify and swiftly treat the complication to avoid lethal consequences. If prompt medical intervention is not provided to the patient, a perforated marginal ulcer could progress to sepsis and death.

## Conclusions

The case describes a patient who presented with at least two major risk factors that exposed and increased her risk of developing marginal ulceration - extended smoking history and the use of non-steroid anti-inflammatory drugs. She presented with unspecific symptoms that were not definitively diagnostic for MU. Still, we could narrow the differential diagnosis with imaging and a detailed history of past medical and surgical encounters. After surgical repair with a Graham patch, our patient was released home after five days with no complications. The value of preoperative counseling for gastric bypass patients cannot be overemphasized as it helps to identify and reduce risk factors to keep complications to a minimum. 

Although rare, marginal ulcer perforation is still one of the most common and severe complications of gastric bypass surgery. They can occur right away after the procedure or present years later. Over-the-counter pharmacological therapy and preop prophylactic treatment have effectively treated ulcers before perforation. However, in some cases, as discussed earlier in this paper, marginal ulcers can develop into perforating ulcers and may require surgical intervention. An exploratory laparotomy was the best-suited technique that enabled the team to identify and treat the perforation before further damage occurred. 

## References

[1] Seeras K, Acho RJ, Lopez PP (2022). Roux-en-Y Gastric Bypass Chronic Complications. https://www.ncbi.nlm.nih.gov/books/NBK519489/.

[2] Chung KT, Shelat VG (2022). Perforated peptic ulcer - an update. World J Gastrointest Surg.

[3] Spaniolas K, Yang J, Crowley S, Yin D, Docimo S, Bates AT, Pryor AD (2022). Association of long-term anastomotic ulceration after Roux-en-Y gastric bypass with tobacco smoking. JAMA Surg.

[4] Madura JA 2nd, Dibaise JK (2012). Quick fix or long-term cure? Pros and cons of bariatric surgery. F1000 Med Rep.

[5] Alkhafaji B, Khafaji YA, Younis MU (2022). Laparoscopic repair of perforated marginal ulcer after Roux-en-Y gastric bypass: a case report and review of literature. Minim Invasive Surg Sci.

[6] Steinemann DC, Bueter M, Schiesser M, Amygdalos I, Clavien PA, Nocito A (2014). Management of anastomotic ulcers after Roux-en-Y gastric bypass: results of an international survey. Obes Surg.

[7] Felix EL, Kettelle J, Mobley E, Swartz D (2008). Perforated marginal ulcers after laparoscopic gastric bypass. Surg Endosc.

